# Remarks, Gleanings, and Extracts

**Published:** 1874-07

**Authors:** 


### Remarks, Gleanings and Extracts.

BY T. CURTIS SMITH, M.D., MIDDLEPORT, OHIO.

*The Anticipation of Post Partum Hæmorrhage.*—Dr. Atthill has an article on this subject in the *British Medical Journal* of Nov. 1, 1873.

After enumerating the causes which commonly produce *post-partum* hæmorrhage, viz.:—Exhaustion of the uterus, exhaustion of the nervous system of the mother; the too rapid emptying of the uterus, whether as regards the fœtus or placenta; the failing to give due support to, and the injudicious manipulation of, the uterus after the expulsion of the placenta, he discusses the treatment of hæmorrhage arising from either of these causes. Throughout the article, he deprecates the indiscriminate and too implicit reliance upon ergot, and concludes as follows: "Give ergot in cases of labor in which you suspect that *post-partum* hæmorrhage is likely to occur, but do not rely on it exclusively. When symptoms, indicating that the power of the uterus is flagging, show themselves, prevent that exhaustion becoming excessive by the use of the forceps; when you do use them, use them as *aids* to the uterus, not as *substitutes* for its action. Give the uterus time to contract after the birth of the child, and to throw off the after-birth, keeping up in the interval firm pressure on the fundus with the left hand; then support the abdomen by the good old-fashioned binder, and the risk of *post-partum* hæmorrhage will be small. I do not mean to say that by the adoption of this or any other treatment *post-partum* hæmorrhage will be entirely averted; that is impossible, but the risk of its occurring will be reduced to a minimum."—*Boston Med. and Surg. Journal.*

*Effects of Pressure on the Fœtal Head.*—Dr. J. Mathew Duncan reports a case in which persistent digital impression was produced on the parietal bone of a fœtus during labor by the finger of the accoucheur, who was endeavoring to effect artificial rotation. There resulted from this slight, short, but frequently recurring epileptsform seizures, which lasted for a time after the digital impression had disappeared, and which were finally superceded by choreic movements.—*British Medical Journal.*

*Petiteau and Isard on Iodoform as a Topical Remedy.*—Dr. Courteaux (*Annales de Dermatologie et de Syphiligraphie,* vol. v. 1873–4) gives a summary notice of two recent works by these gentlemen on the use of iodoform. Petiteau's conclusions, which Dr. Courteaux says, agree with his own and those of several sugeons at the Midi Hospital of Paris, are as follows:

1. Iodoform is locally anæsthetic.
2. When dusted on ulcerating surfaces, it causes them to cicatrize rapidly.
3. It is most useful in small atonic wounds, or such as tend to creep or enlarge; soft chancres, suppurating buboes; syphilitic, varicose, scrofulous and cancerous ulcers.
4. It is the surest remedy to procure prompt cicatrization of syphilitic ulcers of all kinds.
5. It may be applied as an ointment, or as a solution in glycerine and alcohol, and in these forms is preferable to the powder when there is abundant suppuration.
6. It should be always accompanied in syphilitic affections by internal treatment.

Petiteau attributes its good effects to the simplicity of the dressing, to its antiseptic power, and to the absorbent property it possesses as a powder, and to its giving off iodine freely.

Isard, while agreeing with many of the conclusions of Petiteau, maintains that phagedæna is not controlled by iodoform.—*London Medical Record.*

*Phytolacca Decandra in Skin Diseases.*—We have found the saturated tincture of this common root a most effectual remedy in chronic skin diseases, in from fifteen to twenty drop doses, repeated three times a day. In two cases of the scaly variety, it was successful after arsenic had failed, after a fair trial. Moreover, the chachectic condition of the system in each case seemed to be completely removed, and a healthy state substituted.

In three cases of obstinate acne, it has also effected relief. In the first case, it was given for the relief of chronic muscular pains, the patient being also well spotted with the eruption. After its use for three weeks, both difficulties disappeared. This led to its use in the other two cases, in both of whom the acne disappeared in about two weeks.

*Atropia in Hydrosis of Phthisis.*—Our expectations of this remedy in this troublesome symptom has been more than realized. In a number of cases, it has not only checked the profuse secretion promptly, but with a reasonable degree of permanency. The $\frac{1}{120}$th of a grain twice a day is generally sufficient, and much less will often succeed admirably.

*Tannate of Quinia.*—A correspondent in the *Boston Medical and Surgical Journal*, in reply to your correspondent "Inquirer," I think there is good ground for stating that the tannate of quinia comparatively tasteless, because comparatively insoluble, but that it is not absolutely so. In Kerner's experiments (*Archiv für die Gesammte Physiologie*, iii.), he found that while muriate of quinia, injected subcutaneously, made its appearance in the urine in fifteen minutes, and the sulphate rubbed with sugar and given by the mouth in forty-five, the tannate appeared only after three hours, and then in small quantity. The kinovate of quinia, which is probably the salt actually existing in the bark, was as slowly, but rather more completely, absorbed, while the alkaloid in kinovate of quinidia and in the powdered royal cinchona bark did not appear until even later. It would be fair to deduce from this the rule that if quinia is given either for its local action in the stomach, or in order that it may be absorbed rapidly, or in large quantity, the tannate is a highly ineligible form of administration. It is true of quinia, as of many other substances, that comparative insolubility or "incompatibility" out of the body does not prove inertness in the intestinal canal. Where, however, the amount of the drug to be present in the blood at a given time is of consequence, as in the present case, the form of administration should be carefully considered.

Whether the tannic acid of coffee differs in its action upon quinia from the officinal drug, I do not know.—*Boston Med. & Surg. Jour.*

*Nitrite of Amyl in Epilepsy.*—By J. Crichton Browne, M.D. A striking contrast was found between the susceptibility of general paralytic and epileptic patients, in regard to susceptibility to the action of the nitrite of amyl, the latter being much more strongly affected. By causing epileptics, who had daily attacks, to inhale the drug about the time the attack was expected, it was found that the fit was averted. Good results were also obtained in the status epilepticus, eight patients out of ten recovering after the nitrite was used. This brief summary is far from doing justice to the paper. Cases are reported in full, with the result of the administration of the nitrite, both by day and by night; also, cases in which it was withheld where it had, at first, seemed to avert the fit, and the fit came on.—*Boston Medical and Surgical Journal.*

*Pyœmia.*—Prof. v. Nussbaum recommends burning the bone and its marrow with an iron heated to a white heat after amputations of the limbs, in order to prevent the occurrence of pyæmia. He has employed this method with success nine times during the past six months, pyæmia prevailing in the wards quite as extensively during this time as in the previous year, when two-thirds of the amputated patients succumbed to this disease.—*Communication to the Gesselschaft fur Chirurgie.*

*New Drugs.*—Two new plants, called *Echisis scholaris* and *Garcinia mangostana,* both natives of the Philippine Islands, have attracted some attention at the Vienna Exposition. From the bark of the *Echisis,* a hygroscopic bitter principle has been obtained, to which the name *Ditain* has been given. This has proved not only a substitute for quinine, but it has also been found that in its use the frequently unpleasant after-effects of quinine are avoided. The *Garcinia* has been shown to be a safe, rapid and thorough remedy for dysentery and chronic diarrhœa, as well as all catarrhal affections of the bladder and urethra. Both drugs have been extensively employed in the hospitals of Manilla, under the direction of Professor Miguel Zina, chief physician of that province.—*Zeit. des. altg. Apoth. Ver.*—*Boston Medical and Surgical Journal.*

*Abortive Treatment of Furunculus.*—According to several observers, as recorded in the French journals, the following method "never fails to take effect:" As soon as there is perceived that characteristic redness, round in form and variable in size, with a culminating point in the center, which, red at first, soon turns to a grayish white, dip the finger into a little camphorated alcohol, and gently rub the suspected part, especially the middle; repeat this eight or ten times for half a minute each time. After this, cover lightly with camphorated oil. It is rare for the furuncle to resist repeated applications and sometimes they disappear after the first.—*Boston Medical and Surgical Journal.*

*Hypodermic Injection of Whisky.*—A man who had his foot and ankle crushed by a car-wheel recently, was taken to the Bellevue Hospital. He had lost so much blood, that it seemed doubtful whether he would rally sufficiently to permit amputation. Among the means used to rally his strength whisky hypodermically administered. These proved useful by stimulation, and that, too, at a time when gastric irritability caused him to reject everything he would swallow. The pulse was raised sufficient to warrant amputation of the leg, which was done after applying Esmarch's apparatus.—*New York Record.*—*Clinic,* February 28.

*Chronic Bronchitis Treated by Incense.*—The latest "wrinkle" in medication is the invention of an English firm, Field & Co., of Lambeth. They make candles containing in their substance some of those gumresins and balsams, especially benzoin and storax, which from time immemorial have been found useful in chronic bronchitis and allied maladies. When burned, the candles yield, by the combustion of these drugs, a pleasing fragrance, and at the same time give a fairly good light. The idea is a good one.—*Medical and Surgical Reporter.*

### REMARKS, GLEANINGS AND EXTRACTS

BY LOCAL EDITORS.

*Cough Remedies.*—Dr. J. M. Scudder (*Eclectic Medical Journal*) thus discourses: In the early part of my practice I had a "cough medicine," made after the following formula: ℞. Tinct. asclepias, camphorated tinct. opium, sirup of squills, simple sirup, aa., and it did seem like a panacea; at least one would think so from the frequency of its prescription. But a few unpleasant failures awakened me to the importance of selecting my remedy with reference to the condition of the patient.

Cough is the expression of irritation of the sensitive nerves of the respiratory organs. The pneumogastric is the principal, and only in exceptional cases do we have cough from a wrong of the sympathetic. As the pneumogastric has a wide distribution, and irritation in any part may produce cough, cough is not always an expression of diseased lungs. We may have cough from a wrong of the stomach, of the intestinal canal, of the liver, and also a wrong of the origin of the nerve in the brain, as in whooping cough, asthma, etc.

Wrongs of stomach, intestinal canal, etc., have more to do with cough than most persons imagine. My old preceptor used to say that cough medicines would never prove effective if the bowels were constipated—always have open bowels if you wished to get rid of a chronic cough—and there was a good deal of truth in it. Not of course, that you must purge your patient in the old-fashioned way, but you want one free discharge daily. Treating a chronic cough of childhood in the olden time, you would be told to give laxative doses of sulphur, and our Prof. Howe would insist to-day that sulphur was the best cough medicine in these cases—and it is good, at least occasionally.

If the wrong is principally at the origin of the pneumogastic, we think of *nerve* remedies, as drosera, belladonna, bromide of ammonium, clover hay, etc. Selecting the remedy according to special symptoms if the present.

If a cough shows the spasmodic character of whooping cough, or the cough of measles, we would give drosera, ʒj. to water, ℥iv.; a teaspoonful four times a day.

If the patient was dull, drowsy, with expressionless face and eyes, I should think of belladonna as a cough remedy. A common prescription includes aconite as a stimulant to the general circulation: ℞. Tincture of belladonna, gtts. x.; tincture of aconite, gtts. v.; water, ℥iv.; a teaspoonful every one, two, three, or four hours, as needed.

If the cough was epileptiform—paroxysmal, with especial want of control over the respiratory muscles, the remedy would be one of the bromides, usually bromide of ammonium. The reader will recognize the difference between a paroxysmal cough in which the wrong is a sense of internal irritation, and one in which the lesion is especially in a want of control of the respiratory muscles—which once becoming engaged in the act of coughing, do not know when to stop. I usually prescribe bromide of ammonium, ℥ss.; water, ℥iv.; a teaspoonful every three or four hours.

Red Clover is an excellent cough remedy, when the wrong is especially nervous, and the cough explosive. We make an infusion of the carefully dried tops and leaves, and give it in teaspoonful doses every two or three hours.

Nitric acid is an admirable cough remedy when it is indicated—it will be recollected as one of the olden time whooping cough remedies. Indications, a *violet* color of tongue, mucous membranes, lips, or veins—but especially of tongue. ℞. Nitric acid, gtts. xx.; water, simple sirup, aa. ℥j.; a teaspoonful every three hours.

Stillingia is a very fine remedy in chronic cough with soreness of throat. Especially in those cases in which there is a sense of rawness with occasional quick darting pain in the pillars of the fauces and the soft palate. It may be prescribed in any form, but I prefer: ℞. Fluid extract of stillingia, ʒj. to ʒij.; sirup, ℥iv.; in teaspoonful doses, slowly swallowed. The stillingia liniment is also a good preparation in these cases—one drop on a lump of sugar, slowly dissolved in the mouth and swallowed.

Lobelia, for its *specific* action, is the remedy when there is præcordial oppression, or a sense of weight and constriction about the base of the lungs. Especially, when there is fullness and oppression of pulse. Where the oppression was marked, I should give a single dose of gtts. v. to x., and follow with: ℞. Tinct. lobelia sem., ʒss.; water, ℥iv.; a teaspoonful every two or three hours. A much smaller dose than this will frequently answer.

Prodophyllin is an excellent cough medicine in some cases. I recall an old formula of a physician in Eastern Kentucky, which was used with much success: ℞. Podophyllin, prunus virginiana, convallaria multiflora, aa.; make a strong infusion, and add sugar to the consistence of a sirup. In the cough of the early times in the West, arising from great exposure, there was usually a wrong of all organs supplied from the solar plexus—stomach, liver, spleen, upper intestine—and this remedy seemed to do just what was necessary. If I was to name an indication for podophyllin now, it would be *fullness of superficial veins.*

Sanguinaria is an excellent cough medicine when there is a sense of *constriction* in the throat. I employ the nitrate of sanguinaria, one grain to the ounce of simple sirup; dose, one-fourth to one teaspoonful.

Bryonia may be employed if the cough is associated with pleuritic pains, but especially if attended with pain in the base of the brain, or right orbit, or dark flushing of right cheek, or is associated with rheumatism of serous membranes.

Rhus is a remedy when the cough is hacking, and associated with burning in the fauces, or respiratory passages—burning in nasal cavities is an indication. Flushing of left cheek, with burning and left orbital pain, are usual indications.

Veratrum is a most excellent cough medicine—not palliative—and of course not speedy in its effect, but curative, as are all these under consideration. Give veratrum when the pulse is *large* and has strength, and also when it is small but the impulse is so indistinct that it is counted with difficulty. Veratrum is one of the best cough remedies in phthisis, as it very certainly is in acute inflammation of the respiratory apparatus.

Arsenic is an admirable remedy in some chronic coughs. Two cases may be named. In one the tongue is large, slick, and presents a peculiar incurvation of edges as if a "quarter round" had been made on its upper surface, and the queerest color—a *leaden* shade of red. In such cases its action is remarkably prompt and certain. The other case shows the inelastic skin, want of tone in pulse, and frequently tawny coloration of surface. Dose, drops one to four three times a day, of Fowler's solution, or sometimes pellets medicated with this will be sufficient.

Nux Vomica is also a cough remedy, and a very good one. Given, a cough with uneasiness or pain about the base of the lungs, with occasional pain about umbilicus, I should give nux. Or if there was the yellow sallowness of surface, and slight yellow coat of tongue.

Apocynum is a capital cough remedy, and will be indicated in quite a number of cases. *Fullness of cellular tissues* is the indication—whether it is œdema of feet, or puffiness of eyelids, or a similar condition of mucous membranes. The dose is small. ℞. Tinct. apocynum, gtts. x. to gtts. xx.; water, ℥iv.; a teaspoonful every three hours.

Collinsonia is a remedy when cough points in the larynx, especially when use of the voice excites irritation and cough. It is also a remedy when cough has its origin in wrong of the stomach.

Cuprum is a remedy when skin and mucous membranes have a *sodden* appearance, and especially if a greenish tinge is noticed in skin, or fur upon the tongue.

Pulsatilla is a remedy when cough is associated with uneasiness, restlessness, and desire to be constantly changing position; when there is dizziness, inability to command the voluntary muscles, or difficulty in swallowing—nervous dysphagia being a marked indication.

These are examples of the direct action of remedies, and the indications for the particular remedy are given as plainly as possible. I generally select from this class, because they are curative, and do not simply remove the symptom cough.

It is hardly worth while to speak of the olden use of expectorants, for the reader can obtain that in any Materia Medica. There are exceptional cases in which the use of a *nauseant* expectorant to establish secretion will be good treatment; as there are other cases where the *stimulant* expectorants will be beneficial in checking too profuse secretion. Still, it is my opinion that cough remedies should rarely be used in gross quantities, and put in the stomach. Probably we would do quite as well to dispense with the class "expectorants."

For palliation, or temporary relief, I like the application of remedies to the throat. The reader will recollect the very close relation between the sensitive nerves of the throat and the pneumogastric, and how frequently a cough points in the throat, arises from tickling there. We may take advantage of this, and will find in practice, that a remedy applied to the throat to influence these nerves, is far more valuable than if put in the stomach.

We commence with a simple demulcent. ℞. Gum Arabic and white sugar, aa. We acidulate it by adding a small portion of citric acid; make it effervecent by adding bi-carbonate of soda and citric acid; add chlorate of potash; alum; tannic acid; finely powdered cubebs; oil of stillingia; oil of lobelia; nitrate of sanguinaria; aconite; belladonna; morphia; squills; senega; or indeed anything that may seem desirable. Here is a broad field for selection, and the method will be found to give most excellent results.

*Cases in which Fullers' Earth Acted as a Successful Surgical Dressing.*—By Frederick William Gordon, A.B., M.D., late Interne Rotundo Lying-in Hospital, Dublin, Ireland.—I beg leave to present the following cases as the result of the earth-treatment, not that I intend to enter into any discussion upon this mode of surgical dressing, as has already been fully and elaborately done by my friend Dr. A. Hewson, of Philadelphia.* The following cases may be of some interest to the profession in New York, as this mode of treatment is not generally resorted to by surgeons

* "The Use of Earth as a Topical Application in Surgery," by Addnell Hewson, M.D., one of the attending surgeons to the Pennsylvania Hospital, 1872.

here, and indeed has only been, I believe, but very imperfectly tried at Bellevue Hospital some three years ago.

Case I.—A. G., a widow, keeping a boarding-house in the city, æt. 33, a native of the United States, had been suffering for nearly two years from a varicose ulcer of the lower third of the left leg, on the inner side. Her appearance when I was called to see her was that of a healthy woman; but her history was imperfect, as she did not choose to be frank and candid with me. She was the mother of three healthy children. She had been attended by some physicians during this time, and all kinds of treatment, constitutional as well as local, had failed to produce any favorable impression on the ulcer, which finally became so painful as to cause her to confine herself to her bed. The patient informed me when I first saw her that she had noticed no great increase in the extent of the ulcer for the past eight months, but that it had gradually grown deeper, and that each day it was becoming more offensive. She had been in the habit of washing it carefully morning and night. When I first examined the leg, it was as large as the palm of her hand, with callous edges, and a grayish, shreddy surface. The veins of the leg were considerably enlarged. At this time she had been confined to her bed for five days, and suffering great pain. I applied, at my first visit, iodoform in powder to the ulcer, strapped the part firmly, and bandaged the leg from the toes to the knee. I continued this treatment for five days, during which time she kept her bed, suffering great pain. The iodoform had the effect of cleaning off the sore nicely, but it showed no signs of healing at this time. The pain, however, which the iodoform produced was most intense, destroying her rest, and causing her continually to complain of burning in the leg. On the sixth day I gave up its use, and applied dry earth. This gave her entire and immediate relief, and she passed the following night comfortably. She felt much refreshed on the following morning, and told me that she had felt no pain whatever in her leg since the application. She was in good spirits, had eaten a good breakfast, and seemed much encouraged. The dressing was then washed off with castile soap and warm water, the ulcer presenting a clean surface, and gave no pain whatever. I then applied earth, moistened with water, in the form of a paste, covered the part with waxed paper, and over this a firm and even strapping and a collar bandage. The patient was ordered to still keep her bed. No pain whatever succeeded the dressing. She rested well that night, and expressed herself as feeling perfectly comfortable on the following morning. On the next day, the fourth on which the earth had been used, I applied it in a fine powder, covered with waxed paper, strapped firmly, and applied the roller. Still no pain in the part. The next morning, at my visit, I found that a bridge of tissue was forming across the centre; finely-powdered earth applied, and the dressing continued as before. Sixth day: bridge across the centre complete, and the part cicatrizing rapidly. The treatment was continued. On the eighth day of the use of the earth, the patient begged to be allowed to leave her bed and occupy her lounge. I consented to this, and nothing unfavorable resulted from the indulgence; treatment continued. On the tenth day the ulcer was reduced to a small point, and under the daily application of the dry earth, dressing, washing, strapping, and bandaging, on the thirteenth day the cicatrization was complete.

CASE II.—J. F., a tinsmith, æt. 47, came to my office and showed me a varicose ulcer of the left leg, of seven weeks' standing. The veins of the leg were considerably enlarged. The ulcer was much inflamed, and very dirty. The patient was well nourished and healthy in appearance. He denied ever having had syphilis, but confessed that he had had gonorrhœa some ten years before. He was not intemperate, nor would he acknowledge himself guilty of any great excess. He was the father of two children, who had always enjoyed good health. I applied a dressing of wet earth, strapped the ulcer firmly and evenly, and a roller bandage from the toes to the knee; told him to go home and keep his bed. On measurement, the following morning, I found the ulcer to be $1\frac{3}{8}$x$\frac{1}{2}$ inches. It was washed with castile soap and warm water, and dry earth, finely powdered, applied, strapped and bandaged as before. The ulcer had been giving the patient previously so much pain that he willingly had consented to give up his work and remain in bed. The pain had entirely ceased after the application of the earth; and on the following day I found him sitting up, and very comfortable, and feeling able, he said, to go back to his work. I kept him quiet, however, during the following four days, until, under the dressing and treatment, the cicatrization was complete.

CASE III.—Mr. J., from Boston, sent for me to visit him at his hotel (Hoffman House), and showed me a carbuncle which had been about two weeks developing itself. He was suffering intense pain, and was unable to obtain any sleep the previous night; indeed, he told me he had been able to obtain but little sleep during the entire time of its development. He had been applying poultices to it for more than a week. He did not claim to be a temperate man, but usually enjoyed good health. There was some pus coming out of two openings in the centre when I first saw him. The induration of the cellular tissue was considerable at its base. I made a crucial incision through the carbuncle, down to the healthy tissue beneath, without the use of any anæsthetic, and filled the wound with finely powdered dry earth. After the agony caused by the incision, the relief this application gave was very great. The patient slept soundly that night, and was sitting up on the following morning when I visited him. The earth was then washed off. The slough was seen to be protruding, and the tissues were contracting vigorously. I reapplied the dry earth, and retained it by a covering of waxed paper and a few straps. On the evening of the third day after the incisions were made, they had greatly diminished in extent. Dry earth was reapplied, and the wound dressed as before. On the fifth day I removed a portion ot the core, as it was projecting, and dressed the wound with dry earth as before. On the seventh day, a clean excavated ulcer occupied the position of the carbuncle, and four days after, the surface had filled up to the level of the surrounding tissue. Cicatrization progressed rapidly under the same kind of dressing, and four days later the patient returned to Boston with all evidences of his former trouble removed, with the exception of a small granulating spot, which disappeared a few days after the patient reached his home.

*Notes on Practice and Peculiarities of Treatment:* (in Charity Hospital, New York): *Pneumonia.*—The remedies commonly employed in this hospital in the treatment of pneumonia are quinine, carbonate of ammonia, and the alcoholics; occasionally, if the fever is too brisk, liq. ammon. acetatis is administered. The oil-silk jacket is uniformly adopted. Quinine is administered from the beginning. Alcohol, as a rule, is early resorted to. Carbonate of ammonia comes in before the second stage becomes completely developed, and is continued throughout the remaining portion of the course. Diet includes hospital extras. An effort was made by one of the visiting physicians to withdraw, somewhat at least, from this highly tonic and stimulating plan of treatment. Accordingly, liq. ammon. acetatis and tincture of aconite were recommended as the chief remedies to be employed during the earlier part of the disease; but the experiment proved so disastrous, the rate of mortality increasing so rapidly, that the attempt at reformation was at once abandoned.

The constitutional condition of the patients who find admission to this hospital doubtless has a controlling influence upon the treatment necessary to be adopted in this class of diseases, if the best results would be obtained.

*Expectorant Mixture.*—An expectorant mixture very commonly used in cases of chronic bronchitis, and with very good results, is the following: ℞.—Ammon. muriat., liq. morph. sulph. (Mag.) aa ʒi.; syr. tolu, syr. scillæ co., aa ℥i. S. ʒi. t. i. d.

*Night Sweats of Phthisis.*—House-Physician Smith remarked that two-grain pills of oxide of zinc, t. i. d., has answered a better purpose in his division for controlling this symptom than any remedy that had been employed.

*Acute Articular Rheumatism.*—Dr. Smith also directed my attention to an external application to be used for the joints during the progress of this affection. The following is the formula: ℞.—Tr. opii, ℥ i.; spts. chloroform, ℥ iss.; lin. saponis, ad. O i. M. This liniment is applied freely over the joints, and immediately covered with cotton and oil-silk. The relief from pain afforded by this application has been very gratifying to all his rheumatic patients. The general treatment is alkaline.

*Irritable Stomachs.*—The case to which my attention was directed was one in which the ordinary irritability of stomach associated with phthisis required special treatment. The method of treatment is, however, almost uniformly adopted when an irritable condition of the stomach manifests itself in connection with *any* chronic disease.

The remedy is *raw beef*, chopped fine and seasoned with salt, pepper, and vinegar. The patient is to subsist entirely upon beef prepared in this manner. Dr. Smith remarked that this plan had, in his wards, seldom failed to afford relief to this condition, when associated with any chronic affection.

*Silicate of Soda in the Treatment of Fractures.*—House-Surgeon Pierce informed me that he had employed the silicate of soda in his division in the treatment of fractures equally as much as he had employed plaster of Paris. The soda splint has furnished very pleasing results, and, when carefully applied, makes a most elegant and serviceable splint. Three bandages are ordinarily used, the limb being coated over with the silicate after the application of each bandage. It is also well, and perhaps always advisable, to add narrow strips of pasteboard as the bandages are being applied. Extension, in the proper direction, must be maintained until the splint is thoroughly dried.

*Acetic Spray in Diphtheria.*—Diphtheria, scarlet fever, typhus and typhoid fevers, and small pox, constitute a group by themselves.

By present arrangement, this department falls under charge of the hospital staff, as one of the branches of "our-door service." Dr. Partridge, house-physician, mentioned that with regard to diphtheria very satisfactory results had been obtained in the local treatment of the disease by the use of acetic acid, in solutions of varying strength, in the form of spray. The remedy is used by means of the so-called atomizer. It seems to have the power to dissolve the membrane, and in several cases where well developed and somewhat croupy symptoms were present, all were relieved, and that quite speedily, by the use of this agent. The administration of alcoholics is governed by the condition of pulse and temperature. The rate of mortality is small.

*Itching and Pitting in Small Pox.*—To relieve the intense itching which attends this eruption, washing the surface with glycerin and water acts as if by magic.

To prevent pitting, one of the visiting physicians recommended the use of tr. iodine. The remedy should be employed, if possible, before vesicles are formed. It is to be applied once a day. The effect of this remedy has not been sufficiently noted in the Small Pox Hospital to warrant any conclusions relative to its value in this direction.

It was used in one case after the eruption had been vesicular for one or two days, but before it had become pustular, and only a moderate amount of pitting followed. Whether the adoption of an *ectrotic* plan of treatment will not do the patient more harm than can be counterbalanced by the benefit arising from a moderate arrest of pitting, or even a complete prevention of pitting, is, in many cases, thought to be a question worthy of consideration.

To prevent the formation of abscesses, the combined hypophosphites have served a very excellent purpose. One patient had eight abscesses, and another four, at various situations upon the body, and all had rapidly healed under the influence of this combination treatment. In several instances, threatened formation of abscess had been dispelled. The influence of this remedy, therefore, was looked upon with favor, for the reason that abscesses under such circumstances are not unfrequently attended with *grave* results.

"*Treatment of Diabetes Insipidus.*"—"M. Gueneau de Mussey, in a clinical lecture at the Hotel Dieu, recommends the administration of full doses belladonna, and sulphurous baths, in its treatment. He has twice found belladonna to accidentally produce anuria. Its use in incontinence of urine is well established. Systematically employed in diabetes insipidus, it has diminished the quantity of urine passed from ten pints to two pints per diem. The sulphurous baths bring the skin to the relief of the kidneys."

The above query I made myself. Having been troubled for a long time by this disease, at different periods, and it having yielded quite readily, heretofore, to a continued alkaline treatment of carb. soda, I was very uneasy and much alarmed at finding it this time resisting, not only that, but every other remedy authoritatively recommended, and it was gradually reducing my strength and impairing my general health. After writing the above query, and reflecting upon the matter, I had a very dim recollection of an article previously published in your journal on the subject. Upon investigation I found the article above published on the treatment of diabetes insipidus, and determined at once to give it a thorough trial. Having no convenience for sulphurous baths, I adopted the following formula: ℞. Ext. belladonna, grs. xviij; ft. pil., No. xxxvi. S. Take one three times a day.

I took them, with the following physiological effects:

After three days I felt some dryness of the fauces, but no dilitation of pupils; after continuing them eight days, copious evacuations of the bowels, but no abatement of the diuresis. On the twelth day, after taking a brisk walk, feeling badly, and some impairment of vision, I immediately returned to my room, and looked in the glass; pupils were much dilated, and feeling very sick, I took at once about one half grain of morphine. Awoke in the night, found my friends round my bed in considerable alarm, thinking I had been drinking to excess. I told them I was feeling unwell; I had taken some morphine, and I feared an overdose. During that night and till night next day I had a complete ischuria. Commenced diuretics, nit. pot., squills, spts. ether, nit., and digitalis, but for six days the secretion did not exceed six ounces in the twenty-four hours, very high colored and apparently very thick. I had no fever, no pain, no appetite, but a general uneasiness. I could not lie in bed or sit up more than fifteen minutes at a time, and felt, as patients have described their feelings to me, as though I was going crazy. This condition of poison from urea, or belladonna, lasted two weeks. At last, fearing constantly coma and death, I sent for a bottle of Wolf's schiedam schnaaps, and commenced on it with about two ounces; in half an hour repeated the dose; in an hour more urinated freely. It gave me so much relief and I was so much elated, although I had not been out of two weeks, I mounted my horse and rode to a neighboring town, fourteen miles distance, and in the trip finished my bottle of schnaaps. The next day I was sick from the overdose of gin, but urinated freely, and in a few days it assumed its natural color, and has since been voided to the amount of from ten to fourteen ounces pretty regularly every day. I am disturbed only two or three times during a night, and that, I think, more from habit than excess of urine. I conclude in this case belladonna has effected a very satisfactory cure.

*Kumys in Chronic Diarrhœa* (Prof. Stern, of Vienna: *Wierne Mediz. Presse*, April 13, 1873.)—Since the kumys question is fairly afloat, I believe it is the duty of every physician to contribute all in his power which may aid in throwing light on the subject; hence I have been induced to publish the following cases, which I believe contain a certain value:

1. I. K. P., thirty-five years old, has for four years shown a marked tendency towards gout. He has already gone through two attacks of this disease; the first, three and a half years ago, lasted fourteen days; the second, two and a half years ago, lasted five or six weeks. Both attacks were introduced by severe dyspepsia, the pain setting in several days after the dyspeptic symptoms had been relieved. After the first attack, a diarrhœa set in, which was very hard to check. The second attack of gout has, however, remained in all its violence. With care and regulation of the diet, perhaps as much as fourteen days would elapse without any diarrhœa. At the end of this time the trouble would always return with its old violence. As long a period of freedom from the diarrhœa as from two to three weeks could be obtained by drinking constantly red wine, and the use of the "cold water cure." At last, even these lost their power. I then ordered the patient to drink Stahlberg's kumys, at first a small bottle daily, then a large bottle of the strongest variety. In two weeks, the patient told me that the kumys agreed with him, that his diarrhœa had ceased, and that his alvine evacuations were regular. He began this treatment in January of this year, and continued it altogether five weeks. There has been no return of the diarrhœa.

The second case is still more important. Mrs. C. S., thirty years old, has been since her childhood subject to repeated attacks of diarrhœa, which often came without any apparent cause. She was obliged to regulate her diet, and to take every precaution to prevent the recurrence of these attacks. Relief was thus obtained for short periods; but at last the attacks increased in number and intensity, until the patient often was compelled to keep her bed.

In December of the year which has just passed, she consulted me, and I advised her last January to try Stahlberg's kumys, in the same way I had ordered it to the previous patient. In this case, too, the effect was surprising. After using the kumys for four or five weeks, the diarrhœa disappeared, and from the 5th of January until now it has never returned.

*New Treatment of Furuncles.*—Dr. T. N. Wylie, of Bolivar, Texas, (*Medical and Surgical Reporter,*) gives the following case: T. A. P., male, æt. about 25 years; recovered from a severe attack of small-pox in January, 1873, which was followed by a numerous and very painful crop of furunculi. I put him on aquæ chlorini, ℥ss, every four hours, with local application of glycerine and tr. opii., equal parts. There was marked improvement from the first, and complete recovery in about ten days. Has been no return of the furunculi since.

Case II.—W. B. McC. Was called March 7th, 1872. He had been suffering from very painful furuncles, situated on the head and face, for some weeks; at times he was thrown into such violent fever as to compel him to keep his room the entire day; he was put on aquæ chlorini, ℥ss three times a day. The furuncles disappeared in a few days, and have never returned. Four ounces sufficed, in this case, to complete a cure.

Case III.—Is a case of chronic rheumatism, of about eight years' standing, and at times very painful. The hands are prone to erysipelatous inflammation, from scratches or bruises that were received. For this he had been treated with vinum colchici and potas. iodidi, with benefit, but not cured; when he came under my care this treatment had seemingly lost control over the disease. His hands were in a very bad condition when I first saw him; they were very much swollen, pitting deeply by pressure, with large blisters, filled with serum, scattered over the hand. I put him on the aquæ chlorini, ʒvj three times a day, with local application of tr. iodidi for *the first few days.*

I must confess that in this case I was not very sanguine of the result, but from the favorable results of the former cases I was determined to give it a trial.

After the patient had been under treatment for twelve days he came to me with his hands reduced to their natural size, and the chronic sores that had existed for some months entirely well, with one exception, and this one was improving fast (it is entirely well now). In his own words "they are better than they have been in two years." When he receives an injury on them the wound heals as rapidly as on healthy persons; yet, as his is a case of long standing, I shall keep him under treatment for some time.

*Chloral Hydrate in Malarial Fever.*—Dr. T. P. Baily, Georgetown, S. C. (*Charleston Medical Journal*), writes:

I have found it a very useful and important agent in the febrile stage of intermittent and remittent fevers, more especially in those types of a congestive nature.

When the fever heat is intense, its refrigerant and calmative influence is undoubted; and while it quiets the nervous system, it rather promotes the secretions—in this particular being far superior to the opiates. In the malarial fevers of children, with a tendency to convulsion, or when this unfortunate complication has supervened, it can scarcely be excelled. Although we know that the first impression on the gastric surface is rather to excite emesis, nothing can be more demonstrable to me than its promptness in in arresting the vomiting in these fevers when all other efforts have been fruitless. I have frequently stood by the bedside and administered a dose, remaining long enough to note its effect. I have enjoined upon the sick the importance of resisting vomiting for ten minutes, and I do not know of a single instance of its return after the chloral mixture has been retained and kept up during pyrexia. Its rapidity of action does not require its retention long, before we have obtained its expected results.

A few months since I was summoned to a patient who had been vomiting for twenty-four hours, with small, frequent pulse, and all of the signs of capillary stagnation or the congestive termination of fever, and with the use of chloral, which paved the way for other treatment, I succeeded in bringing on a favorable reaction, and afterwards complete apyrexia, which was sustained by the use of quinine. This is one of several similar cases that have not been particularly recorded. With the proper use of mercurials and diaphoretics, made effective by the use of chloral hydrate, I have experienced the most gratifying results.

Patients have suffered almost as much from the secondary effects of opiates as from the original disease. Chloral never produces such results. Under its use the rapid oxygenation of the tissues has been prevented, and a manifest decrease of heat of skin, with lowered frequency of pulse, has been recognized. A patient will speak of the impossibility of making a dose "stick," but if he vomits, it is only an indication to repeat the medicine. He will retain a little, and that little repeated, finally controls the gastric irritation. Of course, a tongue, red and dry, with gastric tenderness, and other special indications, may contra-indicate its use, but a mixture like the following is seldom used without good results: ℞.—Chloral hydrate, ʒ iss.; potass. bicarb., ℈ ij.; spts. æth. nitros, ʒ ss.; sirup tolu, ℥ iiss.; aquæ, ℥ i. M. S. One tablespoonful every hour two, as may be necessary.

Where the cephalalgia and lumbar pains are great, a fraction of a grain of morph. sulph. may be added to each dose of the mixture. The combination seems to prevent the remote unpleasant results of the morphia. It is rare that I use the opiate in combination, as the constipating effects are sometimes unpleasant, and necessitate more medicine. [We give chloral by enema with benefit. G.]

*Treatment of Scarlet Fever.*—Dr. Geo. Bayles, (*New York Medical Journal*) says: As early as possible after the recognition of the disease, and before the fever had attained the extreme of violence, I have derived much satisfaction from the beneficent action of the *hyposulphite of soda.* My friend Dr. M. J. Moses has a formula to which I commonly resort: ℞. Soda hyposulphite, grs. lxiv; syr. tolu, ℥j; aq. cinnamomi, ℥iij. M. Sig. A teaspoonful every two hours. (Two grains of soda hyposulphite in each dose.)

In certain other forms of disease, I consider the hyposulphite of soda as possessing almost the qualities of a prophylactic when employed in time. It is certainly actively eliminative. It is not intended to supersede the fever-powder at any time. They are to be taken conjointly, and at alternate intervals, which will not cause mutual interference.

Where we have diphtheric involvement, I have found much satisfaction in the use of what was suggested by Dr. A. Jacobi: ℞. Acid. carbolic. solut., gtt. x; chlorate sodæ, ʒij; aq. distil., ℥iv. M. Sig. Teaspoonful.

As an agent in the prompt elimination of the poison, and as a general tonic and sustaining medicine, I have a formula suggested by Dr. James R. Leaming, and slightly modified by myself: ℞. Ammon. murias, potass. chloras, aa ʒj; ext. bellad. (English), gr. ss; tinct. ferri mur., ʒj; aq. cinnam, aqua, aa ℥ij. M. Dose, from thirty to sixty drops, repeated every two or three hours.

This medicine, or some essentially similar preparation, cannot be dispensed with throughout the entire course of the disease, even long after the fever, as an objective symptom, has disappeared. It both neutralizes and expels the specific poison, and gives nature a chance to rally on the basis of her own and other judiciously-supplied resources.

In the matter of inunction, I would call your attention to a mixture which seems to have the property of preserving the caloric to a degree not much inferior to that possessed by the butter of cocoa in the direction of its withdrawal. It is of importance to have such a preparation, for there are times when the sudden sinking and exhaustion of the vital powers necessitate the substitution of calorifics for refrigerants. This can only be a temporary condition at any time during the active progress of the disease, but during early periods of invalescence it is often the case that the temperature runs down to a point requiring some prompt and special efforts to induce reaction, and I have found the following formula of great value: ℞. Ol. olivæ, glycerine puræ, aa ℥ijss; solut. carbolic acid, gtt. x; ess. rose-geranium, gtt. xx. M. Ft. lotio.

As a liniment for the throat and chest, as occasion requires, I commonly use the camphorated oil and oil of turpentine in equal proportions, applying the same with cotton-batting and oiled silk.

It not unfrequently becomes necessary to take some measure for restraining the undue action of the bowels. Among the many medicines in ordinary use, I find the tannic acid in mucilage of gum-acaciæ, in proportion to eight grains to the ounce, the most uniformly successful in overcoming the relaxation.

*Cure for Corns.*—A mixture of equal parts of glycerin and carbolic acid, applied with a camel's-hair pencil, is an excellent remedy for these painful companions.—*Journal of Applied Chemistry.*

*Effervescing Solution of Tartrate of Sodium.*—The U. S. Dispensatory says of tartrate of sodium, that it is recommended by M. Delioux as an agreeable purgative, almost without taste, and equal to sulphate of magnesium in its medicinal effects. The merits claimed for the solution of tartrate of sodium are that it is more pleasant to the taste than even citrate of magnesium, while it is more reliable and efficient in its action as a purgative, with less tendency to produce tenesmus. Another decided advantage is the fact of its forming a permanent solution, from which no precipitate settles down, and last, though not least, its much greater cheapness, costing only about one-fourth as much as the magnesium citrate. It would, therefore, seem to be in the interest of both druggists and physicians to make a trial of the new aperient under consideration, which promises to eclipse the now renowned citrate of magnesium.

Mr. Landschutz's formula for filling fourteen of the ordinary twelve-ounce citrate bottles is as follows:

Dissolve nine ounces crystallized tartaric acid, and seventeen ounces crystallized carbonate of sodium, in about one quart of cold water.

Provided the acid is not moist and the carbonate not effloresced, the above solution will be nearly neutral. In general, it is best to test it, and to neutralize it, if necessary. Then dissolve in it twenty eight scruples bicarbonate of sodium. Filter, and add sufficient water to make the entire quantity measure one hundred and forty-seven fluid ounces.

Make a sirup of twenty-one ounces best crushed sugar, fourteen drachms crystallized tartaric acid; ten ounces water. After cooling, add one drachm spirits of lemon, and mix thoroughly. Measure one and a half fluid ounces of this sirup into each of the fourteen bottles. Then pour in slowly the first solution, carefully avoididg an admixture with the sirup; cork and tie each bottle as soon as filled. When this is carefully managed, but very little carbonic acid gas will escape.

Each bottle so prepared will contain about seven drachms of dry tartrate of sodium, which is a fair adult dose.

At present market rates, the above ingredients will cost about five cents for the contents of each bottle, yielding a handsome and remunerative profit. The price, in fact, is so low, that it leaves no incentive towards substitutions, or alterations of the formula.—*A. W. Miller, M.D., Ph.D., in American Journal of Pharmacy.*

*Dyspepsia.*—Dr. Warren, of Boston (*Jour. Gynæcological Soc.*), has employed, with great benefit, the sulpho-binate of sodium in dyspepsia, especially that accompanying pregnancy. He gave it in doses of eight grains three times a day.

*Ice Clysters in the Treatment of Dysentery.*—In my travels during the past and present years, in the position of ship surgeon, to Havanna, New Orleans and New York I had occasion to treat a large number of cases of dysentery both on ship board and on land. Among the various agents to which I was compelled to resort in obstinate cases, one showed itself especially beneficial and at the same time extraordinarily simple, cheap and innocuous in application. So far as I know it has never been used in general practice. I refer to clysters of ice-water or rather of finely powdered ice.

I was led to adopt this treatment in the following way: On the passage from New York I had on the vessel one extremely aggravated case of dysentery. It was characterised by high fever, severe pains in the abdomen and especially by so exceedingly abundant hemorrhages with the very frequent stools that the loss of blood alone involved direct danger to life and induced me to order in symptomatic treatment ice-water clysters every two hours.

A surprisingly happy effect ensued; not only in the fact that the hemorrhages immediately checked up and ceased altogether, but also in the almost immediate abolition of the distressing tenesmus with a reduction of the whole febrile process. I ceased all other medication. The patient himself, so soon as he felt the least manifestation of pain called for ice clysters at once, and under their use alone, this case, one of the worst which I have ever seen, so far recovered in fourteen days that he went ashore at Hamburg with good appetite, etc., in short, in perfect health.

Encouraged with this experience I tried this treatment in less severe and in chronic cases also on myself, for I did not escape attack, when I found that in all the acute and recent cases, light as well as severe, the same excellent results followed. I was compelled to use only rarely small doses of opium in addition. In most cases the medication mentioned was the sole treatment. On the other hand I must say that in chronic dysentery, in old and recurrent cases this means, like all others, is of less or only of transitory benefit.

I am justified in stating, then, that *in all acute and recent cases an energetic local antiphlogosis is most a effective perhaps the most effective treatment of dysentery.*—*Berlin Klinische Wochenschrift*, December 1, 1873.

*Subcutaneous Injections.*—Dr. Constantin Paul recommends glycerin as a dissolvent for subcutaneous injections. He considers it to be far superior to water, alcohol, etc.; it is neutral, can be kept easily, and is, of all liquids, the one which approaches the nearest to the composition of subcutaneous cellular tissue.—*Lancent*, Aug. 2d, 1873.

*Treatment of Orchitis by Nitrate of Silver.*—W. E. Whitehead, M.D., U. S. A. (*Pacific Medical Journal*), says: My treatment of orchitis, either specific or traumatic, has been for several years past as follows: Rest in bed and the application of a thorough coating of a solution of nitrate of silver, from forty to eighty grains to the ounce of water, to the scrotum of the affected side; first having the scrotum well washed in soap and water; the administration of small doses of Epsom salts and tartar emetic dissolved in water, and repeated as often as once in four hours; support to the testicles and low diet.

In some cases it is only necessary to make one, or at most two, applications of the solution of nitrate of silver to the scrotum; but, sometimes it becomes necessary to paint once a day for three or four days before the swelling and pain arrested. When the whole testicle is painful, hot and swollen, my plan is to stretch the scrotum over it gently, and then apply the solution with a camel's hair brush; but, when the testicle is not so seriously implicated, I merely draw the scrotum lightly over the epididimis portion, and apply the solution in the same way. One application I have generally found sufficient to relieve the extreme pain, and at once arrest the inflammation and distressing sense of tension in the testicle, and the lumbar pain that follows the course of the spermatic cord. When the inflammation runs high, I give the tartar emetic in nauseating doses, otherwise in much smaller quantities.

It is necessary during the entire treatment to keep the testicles and scrotum well raised and supported loosely, so as to avoid all strain upon the cord. A well adjusted suspensory bandage will be worn with great comfort for some time after the acute symptoms have been relieved, and the patient is attending to his duties, particularly when he is obliged to be a greater portion of his time on his feet. If the orchitis is specific, all treatment for the specific disease should be stopped at once, for the balsams, injections, and other remedies will only aggravate the new inflammation, and render the recovery much slower.

*Oxygenating Remedies.*—Under this name are embraced several remedies which, when introduced into the blood, lead by their decomposition to a liberation of oxygen. They are best known in this country, in England, and in Belgium; and in the latter, Dr. Van den Corput has made a number of experiments to show their efficaey and demonstrate that the alkaline hypochlorites are to be preferred to the chlorate of potash, recommended by Dr. Smith. Lately Dr. Richardson, of London, has introduced into practical medicine, the use, for this purpose, of peroxide of hydrogen, the dose of which is from half a drachm to a drachm daily. These remedies are indicated in two classes of affections, viz.: those in which respiration is imperfectly carried on, and those which depend upon incomplete nutrition. They render valuable service in asthma, pneumonia, croup, certain heart affections, and are none the less valuable in stimulating the forces which govern nutrition, oxygen being one of the best of tonics.

Dr. Van den Corput has had considerable experience in the administration of a mixture of hypochlorite of soda with cod-liver oil. The latter is administered as an aliment, but when given pure is frequently attended with bad results. When there is a deficiency of oxygen in the blood, owing to pulmonary lesions, it becomes very often injurious to increase the carboniferous elements of the blood without associating with them elements capable of furnishing the oxygen for their combustion.

Dr. Foster, of Dublin, has, on his part, published two cases of occlusion of the foramen of Botal, in which he has been able to show, in the most evident manner, the influence of chlorate of potash and peroxide of hydrogen upon the phenomena of hematosis. In these two infants, the cyanosis was accompanied with evident lowering of temperature, especially in the extremities. Chlorate of potash, in doses of fifteen to twenty centigrammes, caused a diminution in the degree of cyanosis and an elevation of 5° to 6° in the temperature of the fingers. The peroxide of hydrogen, however, produced results even more marvelous, causing the cyanosis to nearly disappear, and raising the temperature of the feet and hands to quite the normal degree. This improvement, in order to be maintained, required the continuous use of the remedy.—*Jour. de Med. de Bruxelles and Lyon Med.*

*A New Method of Producing Local Anæsthesia.*—Dr. Horvath, of Kieff, lately proposed in the *Centralblatt fur die Medicinischen Wissenschaften*, a new method of producing local anæsthesia. If the hand be immersed for a short time in ice water, severe pain is caused. But in experiments made in reducing the temperature of frogs by means of cold alcohol, Dr. Horvath found that no such pain was produced when the hand was immersed in cold alcohol, not even when the temperature of the alcohol was as low as 5° C. Glycerin was found to possess a similar property. Ether caused pain, and quicksilver more acute pain shill, causing the speedy withdrawal of the finger when plunged into this liquid at a temperature of 3°. It was next ascertained that, when the finger was held for quite a long time in alcohol having a temperature of 5° C., no pain was experienced. Moreover, although the faintest touch was distinctly perceived in his finger, no pain was experienced from sharp pricks. This seemed to show that the application of cold alcohol has the effect of depriving the part of the special sensibility to pain without, however, impairing the delicacy of the general tactile sensation, which, as is well known, resides in the superficial integument. This apparent possibility of the artificial separation of these two nervous functions, viz.: the tactile sensation of pain, and the temporary suspension of the latter, seemed important in a physiological point of view, and also of no small practical utility in allaying certain forms of local pain, more especially that caused by burns, and surgical operations. Dr. Horvath had an opportunity of testing the value of this application to burns on his own person, as well as upon others, and not only was all pain instantly allayed, directly the part was immersed in alcohol, but it was found that the wound very speedily began to assume a more healty appearance, the surrounding redness rapidly failing. These observations seem of considerable importance, and we commend them to surgeons both in reference to burns and superficial operations.

*The Treatment of Certain Forms of Bronchocele by Injections of Iodine.*—By Morell Mackenzie, M.D.—In a former paper the author had described in detail the various methods applicable to the several kinds of enlargement of the thyroid gland. In discussing the treatment of fibrous bronchocele in the article referred to, he did not do justice to the method recently introduced by Professor Lücke, of Berne. A larger experience, made under more favorable conditions, had convinced him that the treatment of certain forms of bronchocele by the subcutaneous injection of iodine into the substance of the enlarged gland, was of the greatest value. The following was the plan of treatment, which, in accordance with Dr. Lucke's recommendation, the author had employed: Thirty drops of the officinal tincture of iodine were injected into the substance of the gland once a week for the first two or three weeks, and afterwards once a fortnight, as long as was necessary. It was well to give iodide of potassium internally, at the same time; but no medicine was given to any of the patients whose cases were now related. The advantages of the treatment were, that it did not cause any constitutional disturbance or local irritation (suppuration.) In this respect it was preferable to treatment by setons and caustic darts. The only disadvantage of the method was its slowness; this, however, could scarcely be considered a drawback, except when the the enlarged gland caused urgent dyspnœa.

*How to Solder Broken Files.*—They can be soldered with a common spirit lamp and blow-pipe with common tinners' solder, after first cleaning the broken parts with muriate of zinc.—*Scientific American.*

*On the Treatment of Diphtheria by the Vapor of Iodine.*—Dr. John O'Neill (*Australian Medical Journal*, March, 1873) says the unsatisfactory results of the local treatment of diphtheria have induced him to look afield for some new agent of greater value than those at present in use. He has been led to reject sulphurous acid, whether applied in solution or as vapor from burning sulphur. Iodine in the volatile state has yielded far more satisfactory results. In the form of tincture, iodine has been already long since employed both internally and topically in diphtheria. The author volatilizes twenty to thirty grains of pure iodine by means of a heated shovel placed some little distance from the patient in order to avoid the direct action of the fumes. The fumes are inhaled, and gain easy access to the larynx and trachea. Children seem especially tolerant of the iodine vapor. A milder effect is produced by allowing the iodine to evaporate slowly from flat, shallow dishes. This may be repeated during the day, the object being to keep the air of the room sensibly charged with fumes. The histories of two severe cases are appended. In the one, all the ordinary methods had failed; there had been hemorrhage from the throat, the effusion was extensive, and the patient refused food, and lay in a semi-comatose state. Three fumigations of thirty grains each were employed daily for three days. On the fourth, the exudation began rapidly to clear off. The other case is similar, but in it the membranes seems also to have involved the larynx and trachea.—*London Medical Record.*

*Diseases of the Chest in Children—Their Treatment by Blisters.*—By Daniel Maclean, M.D., Glasgow.—This paper advocated use of, and necessity for, the application of small blisters behind the ear in cases of acute disease, or the acute stage of disease of the chest among children and infants. The author had used this treatment in many appropriate cases, and had found great benefit from its adoption. He founded it on the fact that the nervous system played an important part in all the diseases of the young. Any abnormal action going on in the brain modified the proper influence of that centre upon the tissues at a distance, and gave rise to pathological actions in distant parts. With regard to the lungs, an abnormal action, continuing in a certain part of the encephalon for a time, was conveyed along the efferent fibres of the vagus to their peripheral terminations in the lung-tissue, and stimulated the tissue, and stimulated the tissue to pathological action, thus giving rise to disease in the lungs, from a cause at a distance from the lungs themselves. Again, an excessive irritation of the peripheral terminations of the nerves in the lungs, as in bronchitis, pneumonia, etc., passed along the efferent fibres of the nerves to the brain, and by its continued irritation there became a cause of convulsions, hydrocephalus, etc. This mutual action of nerve-centre and lung-tissue through the afferent and efferent filaments of the pneumogastric nerves, the author held to be the principal cause of the great mortality from chest affections among children. This irritation accounted for the nervous symptoms frequently exhibited by children in these disorders. The greater the amount of nervous sensitiveness and irritation, the greater the danger. For the removal of this important element in chest-disease, the author recommended the use of blisters on oı near the course of the nerves supplying the lungs; a convenient and advantageous spot being behind the ear. This treatment was explained, and the use of blisters generally defended.

*Treatment of Poisoning from Rhus Venenata and Toxicodendron.* James C. White, M.D., Professor of Dermatology in Harvard University (*N. Y. Journal*, March, 1873), has an important paper "On the Action of Rhus Venenata and Rhus Toxicodendron upon the Human Skin," the first being popularly known as poison-sumac, poison-dogwood, etc.; the latter as poison-ivy, poison-vine, etc.

The treatment, when the inflammatory process is well developed, is the selection of applications appropriate to a simple eczema of the same stage. In the great majority of cases, he has found black wash ʒj., lime-water Oj., by far the best application to the affected parts, used as an evaporating lotion upon thin and old linen or cotton cloth for half an hour to one hour at a time, two or three times a day. He has used in connection with it, to moist or excoriated parts, a powder of oxide of zinc ʒj., starch ℥j., or plasters of oxide of zinc or diachylon ointment, as in the management of ordinary eczema. In the black wash, possibly, there may be three elements at work: first, the alkali as antidote, if it is of any avail at such periods; second, the action of cold from evaporation upon the local hyperæmia; and third, the astringent effect of the mercurial powder upon the diseased tissues. In all cases of poisoning, he has been entirely satisfied with its effects, however extensive in distribution or advanced in development the inflammatory condition of the skin. Only upon the thickened epidermal coverings of the efflorescences in the palms does it seem ineffectual. For these special manifestations, he applies solutions of corrosive sublimate, from one to two grains to the ounce of water, in the same way as the black wash is used upon the other parts.

*Wright's Formula for Headache following Alcohol Debauch.*—Take of solution of acetate of ammonia, tinctnre of bitter orange peel, syrup of bitter orange peel, each 20 parts; water, 500 parts. To be given in repeated tablespoonful doses.—*Revue de Therap. Med.-Chir.; Canada Medical Record.*

*Bromo-Chloralum in the Treatment of Vaginal Discharges.*—Dr. Melvin Rhorer, of Louisville, (*American Practitioner*) has been using for some time back bromo-chloralum as a topical application in vaginal discharges with a success which leads him to esteem it an agent of considerable value in this class of cases. Owing to its antiseptic as well as astringent properties, he regards it of especial value in such discharges as are accompanied by fetor. He applies it in certain conditions, undiluted, directly to the seat of the disease by means of pledgets of lint; at other times he uses it as an injection or wash of varying strength, beginning generally with one part of the ordinary solution to eight parts of water.

He says: "Gonorrhea in the female I have also sometimes found to be promptly arrested by this agent. In cases where the inflammation is considerable, and the parts swollen and tender, injections of almost any kind cause, it is well known, great pain. Here before using the remedy it will be necessary first to reduce the inflammation by hot applications, hot hip-baths, injections of warm water where these can be borne, and by constitutional means. Gonorrhea frequently persists by reason of the seat of the disease not being reached by our remedies. It is therefore indispensable to any full measure of success that frequent examinations of the parts involved (the vulva, vagina, and os) should be made, in order to see whether our remedies are being properly applied. Where the disease affects the vulva, vaginal canal, and urethra alone the injections may safely be intrusted to the patient. The same may be said of the management of lint or cotton when used to keep the lips of the vulva apart; but when the disease is deeper seated, affecting the os or the cul de sacs, it will generally be found necessary that the physician should make the injections with his own hand. From a neglect of this precaution cases which had hung on for weeks yielded in a few days."

*Treatment of Erysipelas.*—E. Wigglesworth, Jr., M.D. (*Boston Med. and Surg. Journal*, Feb. 27, 1873), gives the translation of Dr. Kaczorowski's paper on "The Treatment of Erysipelas," published in the *Berliner Klin. Wochenschrift*, Dec. 30, 1872, in which the author believes that erysipelas is an infectious disease, dependent upon the presence of micrococci, a belief justified by the concurrent testimony of Recklinghausen, Waldeyer, Hüeter, Klebs, and Orth. His treatment is, on the one hand, to repress the development and the extension of the globular bacteriums, and, on the other, the support of the resisting power of the organism, the activity of the heart, by stimulation and an easily digestible diet.

To obtain the first result, those parts of the skin which are affected already by erysipelas are rubbed, by means of the finger or a little sponge, every three hours with a mixture of carbolic acid and oil of turpentine (1:10), but very gently; the skin of the neighborhood, however, more energetically. The whole district rubbed is then covered with a soft linen compress moistened with concentrated lead-water (1:100), and finally the parts affected already by the disease are again covered, outside of this compress, by thicker linen cloths dipped in ice-water and lightly wrung out, or by a bladder of ice. The external enveloping compresses with ice-water are changed as often as they begin to grow warm. Internally, the patient takes lemonade or a weak solution of chlorate of potassa, to alleviate the accompanying stomato-pharyngitis and prevent diphtheritic depositions, and every one or two hours a teaspoonful of strong wine, to support the action of the heart.

This treatment has been very effective during the last few months in the epidemic which occurred in Posen, Germany. He has never seen a relapse when this treatment has been pursued.

*A Cure of Epithelial Cancer.*—Dr. Geo. G. Brener (*Philadelphia Medical Times*) says: I have always thought the surgeon's knife was the proper and only treatment for cancer of every description. But my experience in treating an epithelial cancer lately has greatly changed my opinion. The subject of the case was a gentleman fifty years of age, stout and healthy. An epithelial cancer about the size of a hickory-nut located on the cheek near the ear. He consulted other medical gentlemen, who confirmed my opinion and advised him to have it removed. At his request, I removed it with the knife. Part of the wound healed in a few days, but the upper portion soon sprouted out with the cancerous disease. I then applied caustic potassa, not only to it, but to a considerable margin around it. In about ten days after the sloughing was over, I found that the entire margin had taken on the cancerous disease, and my patient was in a worse condition than before the operation. At my request, he consulted several surgeons, who objected to operating any more, for fear of enlarging the cancer, and advised a soothing treatment—poultices of bread and milk. This was followed without benefit for six months, when a friend gave him a recipe which I did not object to his using: Chlor. zinci, gr. viij.; bloodroot, gr. v.; starch, gr. viij. Make into a paste with honey.

The cancer was at this time nearly as large as a hen's egg. After applying the paste for two weeks, he called to see me. I found it had diminished to half its former size. I advised him by all means to continue it. After a month's use of the remedy, the cancer was not larger than a dime. He continued to use it until the disease was cured. There is at this time nothing but a cicatrix, where before was a large epithelial cancer. I report this case for the purpose of calling the attention of the profession to this remedy in epithelial cancer, and do recommend those who have such cases to give it a trial.

*Nitrate of Mercury in Periodical Uterine Hæmorrhages.*—Dr. Joulin has for some time treated the periodical hæmorrhages, which occur between the catamenia, by means of the acid nitrate of mercury, which he applies to the cervical canal by means of a rod of glass moistened with the medicinal fluid. The effect is incontestable, and the new method causes neither pain nor accidents. It is necessary, however, to make a vaginal injection at once, without which there would be severe and persistent smarting. If the cervical canal is contracted, a preparative dilatation is to be made. If the rod does not hold a sufficient quantity of the nitrate, Joulin uses a steel rod on which a little cotton is wound. The little tampon, after having been well moistened with the nitrate, is carried into the cervical canal and then quickly removed. Joulin asserts that he has hardly ever failed in his object by this method, and that he has thus cured hæmorrhages of long duration which had proved rebellious to other modes of treatment. Good results have also been attained in persistent leucorrhœas.—*Jour. de Joulin* and *Gaz. Med. Ital. Lombard.*, No. 35, 1873.

*Cancer*—Prof. W. Parker (*N. Y. Medical Record*) says: I believe that the day must come when something will be accomplished by the aid of internal remedies. Of the remedies now used, arsenic is perhaps the one which commands my confidence more than any other. There is another point in the treatment of cancer which I conceive to be of great importance, and that is the moral condition of the patient. I believe that it is impossible to cure our patients of cancer unless they are buoyed up by hope. Their surroundings should be of a character that will give them the greatest possible amount of comfort and happiness. Keep them in the sunlight of enjoyment, for darkness is the soil in which cancer flourishes.

*Value of Apomorphia in Cases of Poisoning.*—Dr. Loeb relates a case of poisoning of a young man who had swallowed a portion of a solution of 3 oz. oil of bitter almonds in 1½ pint of strong alcohol. Half an hour after the patient was found with a livid countenance, rational, but very weak, vision impaired, pulse 96, heat of body not altered. A subcutaneous injection of 0.008 grm. (⅛ gr.), produced emesis in 8 minutes, which was repeated in 5 minutes. The young man felt better at once, and was well the next morning, with pulse 72.—*Apoth. Zeitung*, 1872, *No.* 45.

*Administration of Strychnia in Nervous Affections.*—Dr Chisolm (*American Journal of the Medical Sciences*, April, 1873) finds that, to obtain all the good that strychia can produce, it is necessary to keep the system under the full physiological effects of the remedy by administering a dose as large as the patient can bear comfortably, and that a smaller dose will not answer. Half a grain *per diem* he considers the maximum dose, but that this could well be borne by most persons. It is better borne when administered after a full meal, and the larger dose in the morning. He finds that a large dose when taken by the mouth will produce the identical immediate and final results as when taken under the skin, and has abandoned hypodermic injections, which he first used. He administers sugar-coated strychnia granules, beginning with seven-thirtieths of a grain three times a day to guard against idiosyncrasies, and gradually increasing the dose to two granules of one-tenth of a grain each after breakfast, and two (or one) after dinner, and one after tea; 'the patient avoids the annoying effects of over-charging the circulation with strychnia, by taking only one pill at bed-time.' By this arrangement the patient takes half-a-grain per day, and the doses may be safely continued for months, or as long as they seem to benefit, and may still further be increased should they cease to excite the nervous centres. The good results are permanent.

*Chloral during Labor.*—Prof. Playfair, of London, strongly recommends the use of chloral in labor, beginning the use of it when the os is dilating and about the close of the first stage. He says it does not predispose to post-partum hemorrhage like chloroform, and tends to relax [illegible] rigid os. He has ʒj chloral prepared in solution of six ounces; gives one-sixth part, or fifteen grains, every twenty or thirty minutes, and frequently the third dose is all that is required. If the case is protracted, he gives smaller doses at longer intervals. He has never needed more than a drachm in any labor. Chloral does not prevent or forbid the use of chloroform if it is needed to lull the strong, forcing pains, but a little will suffice.

*Treatment of Tic Douloureux by means of Ice.*—(W. Wenteritz: *Zur Behandlung des Tic douloureux mit Eisstreichungen—Mitt. des Aerztl. Vereins in Wein*, Bd. 1, No. 1872).—In a very stubborn case of facial neuralgia in a lady, which had not yielded to any of the means applied, a smooth piece of ice was stroked gently over the affected side of the face every five minutes. The painfulness of the application is lessened by holding some alcoholic fluid in the mouth until a slight feeling of warmth is excited. The pain, which disappeared in twelve hours under this treatment, had not returned ten months subsequently.—*Phil. Times, Oct.* 5, 1872.

*Vomiting in Pregnancy.*—Dr. L. Atthill recommends the following formula for the vomiting of pregnancy, and that which sometimes follows severe *post-partum* hemorrhage. From our own experience, we can further recommend it in the vomiting of bilious colic and that caused by biliary calculi. It is used subcutaneously. ℞. Acet. morphiœ, grs. viij.; liquoris atropiæ, m. xlviij.; glycerini, m. iv.; Aqua, ʒ iv. M. Fifteen drops of this solution contain half a grain of the morphia, and about the fortieth of a grain of atropia. It is best, in delicate subjects, to use only from five to eight drops, and repeat if necessary.

Bretonneau strongly recommended belladonna liniment in vomiting, and Guéncan de Mussy also uses the same remedy—combining belladonna extract, one part, with diachylon and theriac plaster, each, two parts; a plaster four or five inches square to be worn on the epigastrium fifteen days if needed.

Dr. T. Bates (*Journal Materia Medica*) advises in scrofulous induration of the nose elixir iodo-bromide calcium comp., a teaspoonful one hour before each meal; after one week, increase the dose to two teaspoonfuls.

This was sufficient to effect a cure, which he pronounces a permanent one, inasmuch as there has been no return of her difficulty since its disappearance, nearly a year since.

He says: I have no hesitation in pronouncing the elixir iodobromide of calcium compound, the most efficient and satisfactory anti-scrofulous preparation I have ever used, but the sphere of its usefulness is by no means confined to scrofula. Aside from this special usefulness, it has an application as diversified as the term alterative can make it.

*Depilatories.*—I call the attention of your correspondent to the assertlon of Dr. Bœtiger, that the best depilatory is the mixture of one part of crystalized sulphydrate of sodium with three parts of fine carbonate of lime, mixed and reduced to a very fine powder. This mixture may be kept any length of time without alteration, in well closed bottles. When moistened with a drop of water and laid, by means of the back of a knife, on the part of the skin covered with hair, in a few minutes the thickest hair will turned into a soft mass, easily removed by means of water.—*Med. & Surg. Reporter.*

*Tetanus.*—Dr. Moffat, of Australia, reports the case of a seaman, in whom, from an injury to his hand, tetanus set in, in its worst form. He was given four ounces of brandy at a dose, and half the quantity whenever he showed signs of consciousness, being thus kept "dead drunk" for four days. The tetanic symptoms passed off, and in three weeks he was well.

*The Value of Oatmeal.*—*La France Medicale* informs us that M. Dujardin-Beaumitz, having obtained *a large* quantity of meal from Scotland, has been *experimenting* with it, young children being the subjects of the experiments. He observes that without speaking of the *bouillies* (porridge?) and cakes which the Scoth prepare from the meal, it is employed by them as food for young children, although the form in which it is said to be so used appears somewhat novel to such of us as have been many years absent from "the land o' cakes," namely, a jelly, prepared by macerating a tablespoonful of the meal in a glass of water for twelve hours, then straining through a sieve, boiling till the whole assumes the consistence of jelly, and adding sugar or salt according to taste. According to analysis, 100 grammes of the meal contain gr. 8. 7 of water, 7.5 of fatty matters, 64 of starch, 12.2 of nitrogenous matters, 1.5 of mineral substances, and 7.6 of cellulose, dextrine and loss. Its nutritious value, therefore, as food for children, in regard to azotic or plastic elements, and such as "respiratory," is analogous to human milk, or that of the cow. Besides, it contains more iron than do most of the ordinary articles of food.

M. Beaumitz had fed four newly-born infants on the preparation just described, and in all three with satisfactory results. He considers that in addition to its qualities as food, it acts efficiently against colic and diarrhœa. It enters into the composition of the *syrup of Luther*, which is said to be much used in Germany. M. Gillette, surgeon of the hospital of Melun, has also given oatmeal "combined with cow's milk," to six children, and his experiments have proved how that food may be valuable in cases where the natural supply of milk is deficient. He adds that the nearer the infant approaches its first year, the more does alimentation by oatmeal appear to be *profitable*.

*The Phosphide of Zinc.*—Dr. Routh, of London, says: A very elegant preparation of phosphorus is the phosphide of zinc. My experience with this medicine is very extensive. I have never known it to produce the least unpleasant effect, and have rarely been disappointed in obtaining the full results to be expected from phosphorus in corresponding doses.

The chemical formula of phosphide of zinc is $6Zn_3$, and consequently one grain represents a little more than 1-7th of a grain of phosphorus. The proper dose is, therefore, 1-7th of a grain. I usually prescribe it in cerebral congestion according to the following prescription: ℞. Zinci phosphidi, gr. iij.; ros. conserv. q. s. ut fiant, pill xxx. Dose, 1, *ter die*. Instead of the conserve of roses, gr. x. of ext. nucis vomicæ may be substituted if strychnia is required.—*Medical and Surgical Reporter.*

*Asthmatic Cough.*—Dr. Gobrecht says, in cases of asthmatic cough, due to the irritation of the bronchi and bronchia, he has obtained great relief in administering the valerianate of zinc in from one to two grain doses three times a day. He has also used the valerinate of zinc in other diseases somewhat similar with success. He suggests that perhaps this remedy and quinia in large doses might control the violent paroxysms of coughing in hay asthma.

Dr. Orr reports several cases of hay fever as greatly relieved by the local application of a solution composed of morphiæ sulph. gr. ij, zinc sulph. gr. i, aq. ℥j. He used the atomizer to apply the solution.

The same gentleman has used the bromide of ammonium in the same disease with relief to the patient. When he used the morphia it was given by means of steam.

*Gelseminum in Urethro-Cystitis.*—A. K. Webster, M:D (*Journal Materia Medica*) writes: Within three months I have treated nine cases with this drug; each case was characterized by incontinence of urine, frequent, scanty and painful micturition with symptoms of local urethritis, also great prostration of strength.

No external symptoms were used, and no medicine except the following prescription: ℞.—Ext. gelseminum (Tilden's), ʒij.; bromide potassium, ʒiij.; aquæ menthæ pip., ℥iiss. M. S. Take teaspoonful every three hours.

To their great surprise and gratification, they were relieved in twelve, and were able to resume labor in forty-eight hours. I had used bromide of potassium in similar cases before, with little or no avail.

*Chronic Eczema of the Extremities.*—Dr. MacCallum, of the Montreal General Hospital (*Canada Med. and Surg. Journal*, November, 1872), successfully treated an obstinate case of this affection, the patient, male, being forty-two years old, as follows: A solution of potassa causticia gr. v., to water ℥ij., was used outwardly, being brushed lightly over the eruption once daily. The following mixture was ordered internally: ℞.—Hydrarg. bichlor., gr. iv.; arsenic, min. xv.; acid hydrochlor. dil., ʒiss.; aquæ ad., ℥viij. M. ℥ss. ter die.—*Medical Record.*

*Mettauer's Mixture.*—This valuable mixture should be prepared as follows: ℞.—Nitric acid, water, muriatic acid, aa ℥j. The constltuents are to be united in the order of their numbers. The doses are from seven to ten drops, largely diluted with sugared water, say not less than four ounces, to be taken an hour before meals. After each dose, Dr. Mettauer advises that the mouth should be washed with an antacid mouth-water.

*Syrup of the Bromide of Iron.*—Take of bromine three troy ounces; iron (in the form of wire) 350 grains; distilled water, three fluid ounces; citrate of potash, six troy ounces or q. s.; syrup, a sufficient quantity. Put the iron, with the distilled water, in a flask, and add of the bromine two troy ounces in small portions at a time, allowing the mixture to cool, if necessary, before the addition of each fresh portion. When the odour of bromine has disappeared, warm the mixture gently, until it assumes a green color; filter and add the remainder of the bromine. Transfer the resulting solution of ferric bromide to a porcelain mortar, and add gradually, with constant trituration, citrate of potash, until the color of the mixture changes from brown to green; finally, add simple syrup to make up thirty fluid ounces. The syrup thus prepared corresponds nearly in strength with the syrup of the iodide of iron, and may be administered in doses of twenty to thirty drops. It is elegant in appearance, not disagreeable in taste, and is not incompatible with alkalies or with tannic acid.

*Quinine by the Rectum.*—Quinine will do its work just as well when mixed with cocoa butter and pushed into the rectum, as when taken by the mouth. This can be done with less trouble and discomfort than attends the swallowing of a sugar-coated pill. The doctor, patient and attendants who have once tried this method of giving quinine to children and fastidious people, will be sure to remember and practice in similar cases. There is no way equal to this for administering narcotics when the stomach is weak, or the patient unwilling to take medicine per mouth.—*Detroit Review.*

*Anti-Neuralgic Snuff.*—The *Revista Clinica di Bologna* mentions an anti-neuralgic snuff prescribed with success in cases of facial neuralgia, by Dr. Scriffignano. The base of the snuff is quinine, and its composition as follows: Citrate of quinine, ten grains, very strong, exciting snuff (tobacco), fifteen grains. The medicament is said to act almost directly on the diseased nerve through the ethmoidal thread of the nasal ramus of Willis's ophthalmic, a branch of the fifth pair.—*London Lancet.*

JACOBI'S food for children is prepared as follows: Crack a teaspoonful of barley in a common coffee-mill, then boil it fifteen minutes in a gill of water, adding a pinch of salt. Then strain, and for a young child add one-half as much cow's milk as you have barley-water, and, whilst tepid, nurse from a nursing-bottle. Sweeten slightly with sugar. If the bowels are costive, use oatmeal in place of barley. Keep the bottle clean.—*Philadelphia Medical Times.*

*Treatment of Gastralgia.*—Dr. Joulin strongly advocates, for the treatment of gastralgia (*France Madicale*), 1. A poultice of ice for ten minutes, morning and evening, to the pit of the stomach. 2. A mustard plaster to the same spot immediately on removing the ice-poultice, to be kept on as long as possible. 3. Pounded ice, to be taken morning and evening, a tablespoonful every five minutes for one hour. 4. A mustard bath with two pounds of mustard three times a week. The ice of the poultice is to be chopped up in small bits and enclosed in an India-rubber bag. The ice taken internally must be pounded with sugar, or it may be prepared by an ice-man to the taste of the patient. It must be swallowed down suddenly, and not kept in the mouth, as it would lose its coldness. If a tablespoonful would be too much at a time, as we fancy it mostly would, several teaspoonfuls may be taken instead. The mustard bath Dr. Joulin regards as a powerful tonic.—*Doctor.*

*Pure Phosphorus in Nervous Diseases.*—Dr. W. H. Broadbent, in the *Practitioner*, makes some useful suggestions respecting the value of simple phosphorus in certain nervous diseases. He has tried all the different methods of administering it, and recommends as the best, phosphorus *dissolved* in *oil* or *lard* and *enclosed* in a *gelatine capsule.* He gives about one-thirtieth of a grain after meals. He says that you might as well expect to get the effects of sulphur by giving sulphuric acid, as to get the effects of phosphorus by giving phosphoric acid. As illustrations of this method of using phosphorus, he details its successful application to cases of *epileptiform vertigo*, of *severe neuralgia, atonic dyspepsia, nervous break-down*, from over-work, etc.

*Morphia in Acute Uræmia.*—Dr. Alfred Loomis asserts that morphia can be administered hypodermically by some, if not all, patients with acute uræmia, its almost uniform effects being—1, to arrest muscular spasms by counteracting the effect of the uræmia poison on the nerves centres; 2, to establish profuse diaphoresis; 3, to facilitate the action of cathartics and diuretics, especially the diuretic action of digitalis. It must be given in sufficient quantities to control convulsions, as neither the contraction of the pupils nor the number of respirations is a reliable guide in its administrations. *New York Medical Record.*

*Aconite in Headache.*—Dr. Fothergill (*British Medical Journal*, January 10, 1874) recommends the use of aconite in congestive headaches, attended with great contemporary coldness of the hands and feet. The remedy dilates the peripheral blood-vessels, and so relieves the congested cerebral vessels.

*Mecca Oil.*—In diseases of the bladder, kidneys, and liver, it is of the greatest value; also in chronic diseases of the bronchi. In incipient consumption it is of more value than any other agent with which I am acquainted. Numerous cases, in which patients had coughs, bad appetites, constipation, and hemorrhages from the lungs, have been entirely cured by using the Mecca Oil. In gonorrhœa it is nearly a specific. One drachm taken four times a day will cure every case in from six to twenty days in healthy constitutions, and in the acute form I have known cures effected in three days. As an external application in most forms of skin diseases it is quite a specific, and infinitely superior to tar anointment, zinc, iodide of lead, and other articles generally used by physicians.—*Dr. Paine.*

*Ergotin Injections in Prolapsus Ani.*—Von Langenbeck, of Berlin, announces that he has lately been treating prolapsus ani "with astonishing success," by hypodermic injections of a solution of ergotin (five to fifteen parts to one hundred of distilled water). He replaces the bowel, and inserting the point of the syringe about three centimetres in depth in the cellular tissue, throws in from one to two grains of ergotin. This should be repeated every three or four days, for three or four weeks, any hard fecal masses in the bowels being first removed by a simple injection. As a means of treating a most obstinate and troublesome complaint, this method, sanctioned by so eminent a name, deserves careful repetition.—*Medical and Surgical Reporter.*

*Eczema of the Genital Organs.*—Dr. Montmeja, of St. Louis Hospital, Paris, has recorded some cases of eczema occuring upon the external genital organs, in *La France Medicale*, July 6th. In some of the cases tincture of iodine has been the chief application, and achieved a cure of the disease in two months. Beyond this he has prescribed daily the following as a lotion to allay the itching in affected part: ℞. Corrosive Sublimate, 2 parts; water, 500 parts; alchohol, a sufficient quantity to dissolve the corrosive sublimate. He has observed in many of these cases the vesicles and scales of eczema supervene on an old standing intertrigo in which mechanical irritation has produced its effects.—*The Doctor, August*, 1873.

*Treatment of Alcoholism by Nux Vomica* (*Irish Hospital Gazette*, May 1, 1874).—Dr. Luton has obtained excellent effects from the use of nux vomica in chronic alcoholism where the evil has not passed into the absolutely degenerative stage of tissue-change. In the tremors, and the cerebral, gastro-intestinal, and thoracic disorders, of alcoholism he resorts with confidence to the use of extract or tincture of nux vomica in ordinary doses.

*Ammonia in Puerperal Thrombosis.*—Dr. Richardson has shown that the exhibition of ammonia, in doses varying from ten to twenty drops of the liquor in sweetened milk every hour or half hour, had a powerful effect in preventing the formation of clot, and in some cases dissolving it when formed. The liquor ammoniæ had a great advantage over the carbonate or alkalies generally. These weakened greatly the already exhausted patient, and their elimination was not so rapid. The administration of the liquor might be pushed almost to complete disintegration of the blood globules, and no danger follow, as they will be rapidly reproduced. It is then the remedy to be tried in all cases of thrombosis, puerperal or otherwise.

*Aromatic Tincture of Assafœtida.*—Dr. L. Myers Connor (*Amer. Jour. Pharmacy*) says: This tincture has such an unpleasant smell and nauseating taste, that it cannot be given in every case. The formula offered has been tried, the aromatics being no objection, either in properties or preparation; it can be made at any time, also keeps well: ℞.—Tinct. assafœtida, U. S. P., ℥viij.; tinct. orange peel, U. S. P., ℥ij.; ess. peppermint, ℥iij. Mix. Dose, one and a half to two fluid drachms, without the addition of water.

*Phosphorus in Melancholia.*—Dr. S. W. D. Williams reports six cases of melancholia treated with phosphorus, three of which resulted in recovery, two in partial benefit, and one in entire failure. In the latter case a cure was rapidly effected, after the withdrawal of the phosphorus, by means of opium. In each case the dose was one pill of one-thirtieth of a grain, twice daily, increasing to three or four pills daily. No toxic effects were observed except a slight warmth in the epigastrium.—*Journal of Mental Science.*

*To Check Vomiting from Cough.*—Consumptives and others suffering from paroxyms of cough frequently vomit their food in such paroxyms. Dr. Woilliez recommends swabbing the pharynx before eating with a concentrated solution of bromide of potash. ℞. Pot brom., ʒij; aquæ destil., ʒiv.; warning the patient not to cough for a few minutes after the application. The same treatment is recommended for the vomiting of pregnancy. It is said to be very successful.—*Med. and Surg. Reporter.*

*Means of diagnosticating Lipomata.*—A character peculiar to lipomata resides in the property belonging to all fatty tumors of hardening under the action of cold. When, after the use of ice or the ether spray, in the case of a doubtful tumor, the growth is felt to become harder, the presumption is that the case is one of lipoma. *Lancet*, August 2, 1873, from *Rev. Med. Phot. des Hopitaux.*

*A Rapid Cure for Tape-Worm.*—A. J. Schafish, of Washington, says, *inter alia:* I made no preliminary provisions further than to forbid the patient from taking any breakfast the day I intended removing the worm, and giving him a large dose of Rochelle salts the preceding night. At ten o'clock in the morning he had the following at one dose: ℞. Bark of pomegranate root, ½ ounce; pumpkin seed, ½ drachm; ethereal extract of malefern, 1 drachm; powdered ergot, ½ drachm; powdered gum arabic, 2 drachms; croton oil, 2 drops. The pomegranate bark and pumpkin seed were thoroughly bruised, and, with the ergot, boiled in eight ounces of water for fifteen minutes, then strained through a coarse cloth. The croton oil was first well rubbed up with the acacia and extract of malefern, and then formed into an emulsion with the decoction. In each case the worm was expelled alive and entire within two hours. No unpleasant effects followed.—*The Druggists' Circular.*

*The Best Method of Using Chlorate of Potassium Topically.*—Prof. Gosselin, having experienced the frequently unsatisfactory performance of this remedy when used locally in cases of mercurial stomatitis, etc., has devised the following method, which he recommends strongly: A concentrated solution of the salt, mixed, if necessary, with a little laudanum to quiet any pain caused by the stomatitis, is placed upon pieces of lint and introduced into the gingival gutters on each side of the mouth. These pieces of lint are to be renewed at short intervals during the day, and may be alternated with the salt in the usual form of a gargle—*Ibid.*

*Treatment of Zona by Collodion and Morphia.*—Dr Bourdon, Hôpital la Charitè, after having tried a great many local means for treating the above disease and checking the intense pain, has definitely adopted the following plan: Without opening the vesicles, he paints all the diseased surface with a combination of collodion and morphia—collodion, one ounce, morphia, eight grains. The mixture must be put on pretty thickly. The pain ceases from the second day, and at the end of seven or eight days, when the layer of collodion is removed, all the vesicles have disappeared, and there remains only a slight local redness.—*London Lancet.*

*Intertrigo.*—As chafing of the skin of various parts of the body is quite common at this time of the year, the following formula may prove timely: ℞. Bismuth subnitrat.; Glycerinæ a a ℥ij. M. A few drops of tincture of cochineal may be added, to give the mixture a flesh-tint, and a little water may be added in warm weather. It may be smeared over the opposing surfaces after they have been carefully cleaned.—*Philad. Med. Times.*

